# Supplementary material for: In Silico Designed Multi-Epitope Immunogen “Tpme-VAC/LGCM-2022” May Induce Both Cellular and Humoral Immunity against Treponema pallidum Infection
Source: Vaccines (Basel). 2022 Jun 25;10(7):1019. doi: 10.3390/vaccines10071019 (PMC9320004; doi:10.3390/vaccines10071019)

Supporting information

Supplementary Table S1. List of *Treponema pallidum* proteins selected for analysis.

	NCBI ID	GENE ID	PREDICTED LOCATION	PROTEIN NAME	PREDICTED FUNCTION
**1**	WP_010882178.1	TP_0733	SEC	Outer membrane beta-barrel protein	OprG/OmpW-like ion-channel involved in transport of small hydro-phobic molecules
**2**	WP_014342713.1	FA889_00665	PSE	Hypothetical protein	Outer membrane protein/outer membrane enzyme PagP, beta-barrel domain
**3**	WP_010881878.1	TP_0430 / ntpK	MEM	ATP synthase subunit K	Proton transmembrane transporter activity
**4**	WP_010882306.1	TP_0862 / slyD	PSE	FKBP-type peptidyl-prolyl cis-trans isomerase	Peptidyl-prolyl cis-trans isomerase activity
**5**	WP_010881883.1	TP_0435 / nlpE	SEC	Copper resistance protein NlpE	Copper resistance associated lipoprotein
**6**	WP_010882040.1	TP_0594	PSE	DUF2147 domain-containing protein	Hypothetical conserved protein
**7**	WP_010882416.1	TP_0972 / ftr1	MEM	FTR1 family iron permease	Iron ion transmembrane transporter activity
**8**	WP_010881498.1	TP_0049	PSE	M23 family metallopeptidase	M23 domain-containing metallopeptidase
**9**	WP_010882004.1	TP_0557	SEC	DUF1007 family protein	ABC-type uncharacterized transport system, periplasmic component
**10**	WP_010881746.1	TP_0297	SEC	SPOR domain-containing protein	Hypothetical protein containing a peptidoglycan binding domain
**11**	WP_010882234.1	TP_0789	SEC	Outer membrane lipo-protein-sorting protein	Outer membrane lipoprotein-sorting
**12**	WP_014342788.1	TP_0326 / BamA	SEC	Outer membrane protein assembly factor BamA	Outer membrane beta-barrel assembly factor
**13**	WP_010881537.1	TP_0088	PSE	Hypothetical protein	Hypothetical protein
**14**	WP_010881771.1	TP_0323	MEM	ABC transporter permease	Ribose/Galactose transmembrane transporter activity
**15**	WP_010881783.1	TP_0335	MEM	CPBP family intramembrane metalloprotease	Metalloendopeptidase activity
**16**	AAC65118.1	TP_0126	MEM	Predicted coding region TP0126	OmpW-like ion-channel involved in transport of small hydrophobic molecules
**17**	WP_010882196.1	TP_0751	PSE	Vascular adhesion/metalloproteas e pallilysin	Vascular adhesin/metalloprotease
**18**	AAF45140.1	tprK	CYT	Tpr protein K	*T. pallidum* repeat protein

Supplementary Table S2. Predicted IFN-gamma inducing epitopes. 69 MHC-II epitopes capable to induce IFN-gamma (positive) were predicted using the IFNepitope server hybrid method (MERCI and SVM) default parameters.

Epitope sequence	Method	Result	Score
GTPQNITDLCAEYHN	MERCI	POSITIVE	1
TPQNITDLCAEYHNT	MERCI	POSITIVE	1
PQNITDLCAEYHNTQ	MERCI	POSITIVE	1
QNITDLCAEYHNTQI	MERCI	POSITIVE	1
NITDLCAEYHNTQIH	MERCI	POSITIVE	1
ITDLCAEYHNTQIHT	MERCI	POSITIVE	1
TDLCAEYHNTQIHTL	MERCI	POSITIVE	1
NDKIFSYTESLAGKR	MERCI	POSITIVE	1
DKIFSYTESLAGKRE	MERCI	POSITIVE	1
KIFSYTESLAGKREM	MERCI	POSITIVE	1
IFSYTESLAGKREMA	MERCI	POSITIVE	1
FSYTESLAGKREMAI	MERCI	POSITIVE	1
SYTESLAGKREMAII	MERCI	POSITIVE	1
YTESLAGKREMAIIT	MERCI	POSITIVE	1
EYAFAAYAVHALWNA	MERCI	POSITIVE	1
FVLGPGPGVHALWNA	MERCI	POSITIVE	1
PGPGGIVSFDFFFDA	MERCI	POSITIVE	1
FFFDAAMVYGPGPGG	MERCI	POSITIVE	1
AAMVYGPGPGGQKWT	MERCI	POSITIVE	1
AMVYGPGPGGQKWTY	MERCI	POSITIVE	1
MVYGPGPGGQKWTYE	MERCI	POSITIVE	1
VYGPGPGGQKWTYEL	MERCI	POSITIVE	1
YGPGPGGQKWTYELY	MERCI	POSITIVE	1
GPGPGGQKWTYELYL	MERCI	POSITIVE	1
PGPGGQKWTYELYLE	MERCI	POSITIVE	1
PGGQKWTYELYLEIL	MERCI	POSITIVE	1
GGQKWTYELYLEILQ	MERCI	POSITIVE	1

GQKWTYELYLEILQK	MERCI	POSITIVE	1
QKWTYELYLEILQKG	MERCI	POSITIVE	1
TLFAGAAGAGPGPGG	SVM	POSITIVE	1,0027957
AAVLAAYRAVRTLLI	SVM	POSITIVE	1,021854
SVALFYAYAAYAAAA	SVM	POSITIVE	1,0643286
AAYAAAVTEYAFAAY	SVM	POSITIVE	1,084482
HAIAAISMANEAAAK	SVM	POSITIVE	1,0846252
LFAGAAGAGPGPGGG	SVM	POSITIVE	1,1717537
IAAYAAAVTEYAFAA	SVM	POSITIVE	1,2066697
AYAAAVTEYAFAAYA	SVM	POSITIVE	1,2083039
GAGPGPGGGGGFHLG	SVM	POSITIVE	1,2582538
LIIAAYAAAVTEYAF	SVM	POSITIVE	1,2673741
AAGAGPGPGGGGGFH	SVM	POSITIVE	1,3224505
YAAAVTEYAFAAYAV	SVM	POSITIVE	1,3276033
AGAGPGPGGGGGFHL	SVM	POSITIVE	1,4281056
GAAGAGPGPGGGGGF	SVM	POSITIVE	1,5152442
FAGAAGAGPGPGGGG	SVM	POSITIVE	1,5171639
AGAAGAG-PGPGGGGG	SVM	POSITIVE	1,6719466
YAFAAYAVHALWNAY	MERCI	POSITIVE	2
AFAAYAVHALWNAYA	MERCI	POSITIVE	2
FAAYAVHALWNAYAA	MERCI	POSITIVE	2
AAYAVHALWNAYAAY	MERCI	POSITIVE	2
AYAVHALWNAYAAYH	MERCI	POSITIVE	2
YAVHALWNAYAAYHA	MERCI	POSITIVE	2
AVHALWNAYAAYHAL	MERCI	POSITIVE	2
VHALWNA-YAAYHALW	MERCI	POSITIVE	2
VLGPGPGVHALWNAY	MERCI	POSITIVE	2
LGPGPGVHALWNAYA	MERCI	POSITIVE	2
GPGPGVHALWNAYAI	MERCI	POSITIVE	2
PGPGVHALWNAYAIA	MERCI	POSITIVE	2
GPGVHALWNAYAIAA	MERCI	POSITIVE	2

PGVHALWNAYAIAAA	MERCI	POSITIVE	2
GPGGIVSFDFFFDAA	MERCI	POSITIVE	2
PGGIVSFDFFFDAAM	MERCI	POSITIVE	2
GGIVSFDFFFDAAMV	MERCI	POSITIVE	2
GIVSFDFFFDAAMVY	MERCI	POSITIVE	2
IVSFDFFFDAAMVYG	MERCI	POSITIVE	2
VSFDFFFDAAMVYGP	MERCI	POSITIVE	2
SFDFFFDAAMVYGPG	MERCI	POSITIVE	2
FDFFFDAAMVYGPGP	MERCI	POSITIVE	2
DFFFDAAMVYGPGPG	MERCI	POSITIVE	2
GPGGQKWTYELYLEI	MERCI	POSITIVE	2

Supplementary Table S3: Predicted conformational B-cell epitopes. The ElliPro server was used to predict the conformational B cell binding epitopes via the 3D structure of the *Mycoplasma pneumoniae* multi-epitope vaccine.

Conformational epitope	Residues and position	Number of residues	Score
1	M1, I2, K3, L4, K5, F6	6	0.969
2	P359, G360, P361, G362, G363, M364, F365, G366, A367, A368, A369, V370, L371, G372, I373, S374, A375, V376, G377, G378, P379, G380, P381, G382, K383	25	0.898
3	G7, V8, F9	3	0.859
4	R387, A388, V389, R390, T391, L392, L393, I394, I395, C396, A397, G398, P399, G400, P401, G402, V403, L404, H405, A406, A407, A408, A409, V410, T411, E412, Y413, A414, F415, V416, L417, G418, P419, G420, P421, G422, V423, H424, A425, L426, W427, N428, A429, Y430, A431, I432, A433, A434, A435, A436, R437, G438, P439, G440, P441, G442, R443, P444, A445, G446, S447, A448, T449, L450, F451, A452, G453, A454, A455, G456, A457, G458, P459, G460, P461, G462, G463, G464, G465, G466, F467, H468, L469, G470, Y471	85	0.745
5	F10, T11, V12, L13, L14, S15, S16, A17, Y18, A19, H20, G21, T22, P23, Q24, N25, D28, L29, C30, A31, E32, Y33, H34, N35, T36, Q37, I38, H39, L41, D43, K44, I45, F46, S47, Y48, T49, E50, S51, L52, A53, G54, K55, R56, E57, M58, A59, I60, I61, T62, F63, K64, N65, G66, A67, T68, F69, Q70, V71, E72, V73, P74,	107	0.741

G75, S76, Q77, H78, I79, D80, S81, Q82, K83, K84, A85, I86, E87, R88, M89, K90, D91, T92, L93, R94, I95, A96, Y97, L98, T99, E100, A101, V103, L106, C107, V108, W109, N110, N111, K112, T113, P114, H115, A116, I117, A118, A119, I120, S121, M122, A123

Supplementary Figure S1. Population coverage of the alleles used in the construction of the final version of Tpme-VAC/LGCM-2022, with a total population coverage of 99.93%

MHC class	Coverage	Average hit	PC90
combined	99.93%	16.05	8.08

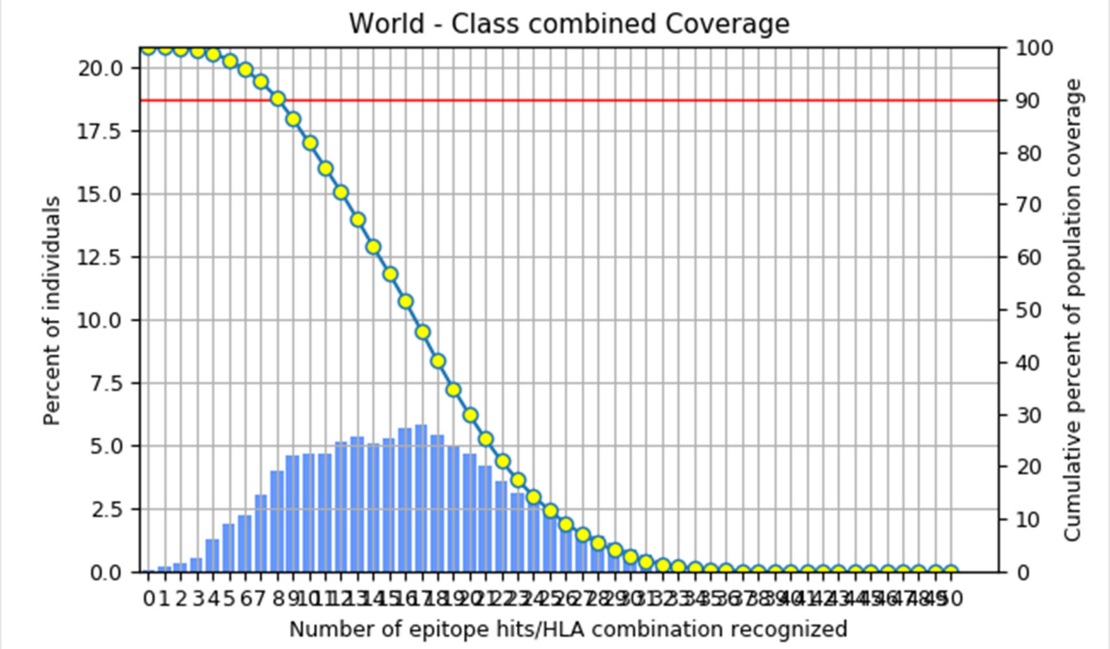

Supplementary Figure S2. Representation of the secondary structure of the chimeric protein. The results showed an arrangement of alpha helices (48.0%), β-strand (15.0%), and coil formation (37.0%).

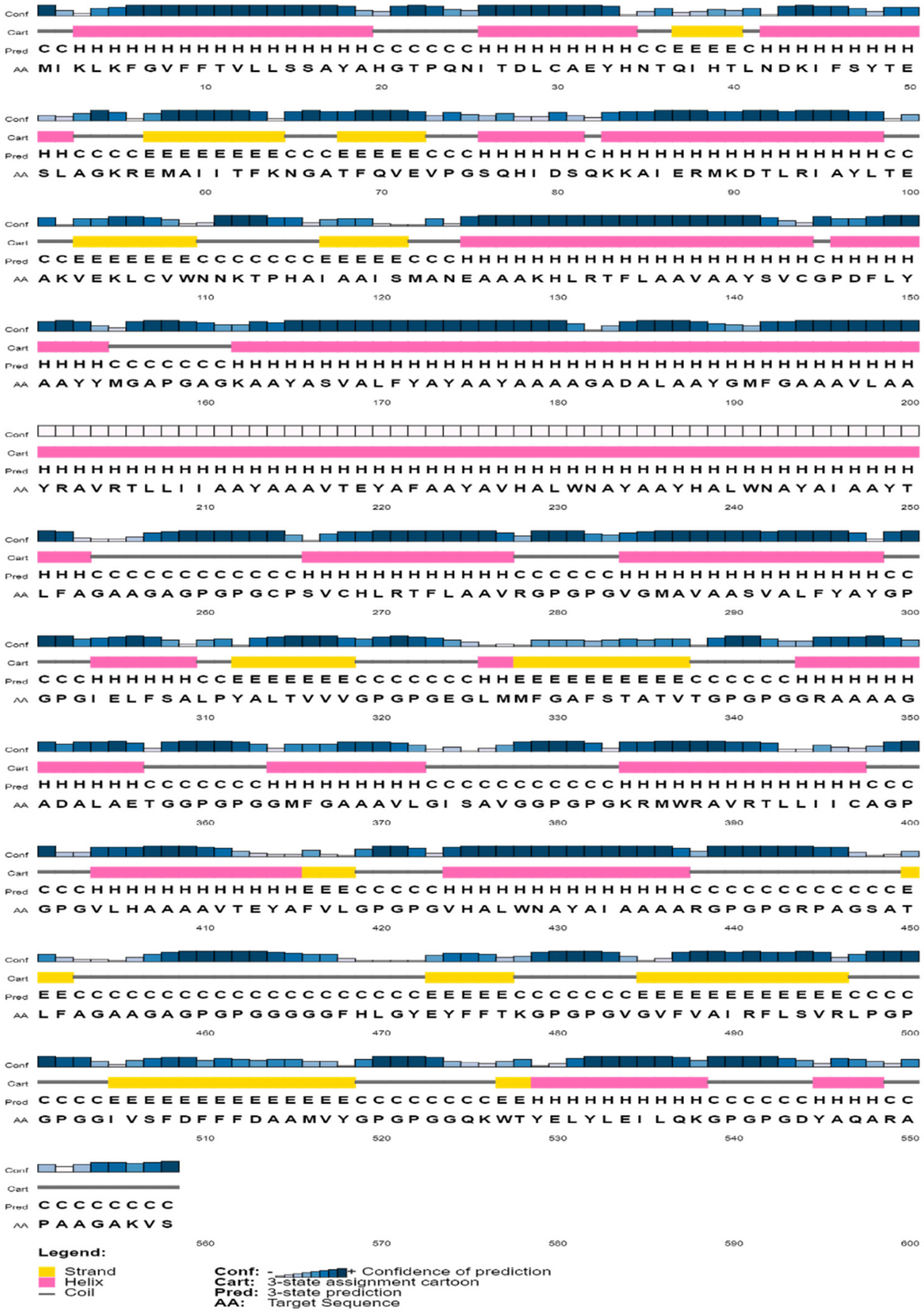

Supplementary Figure S3. Three-dimensional structure modelling of the chimeric protein. (A) Model predicted by RaptorX. (B) Ramachandran plot for the initial model, showing 92,9% residues in most favoured regions and 0,7% in disallowed regions.

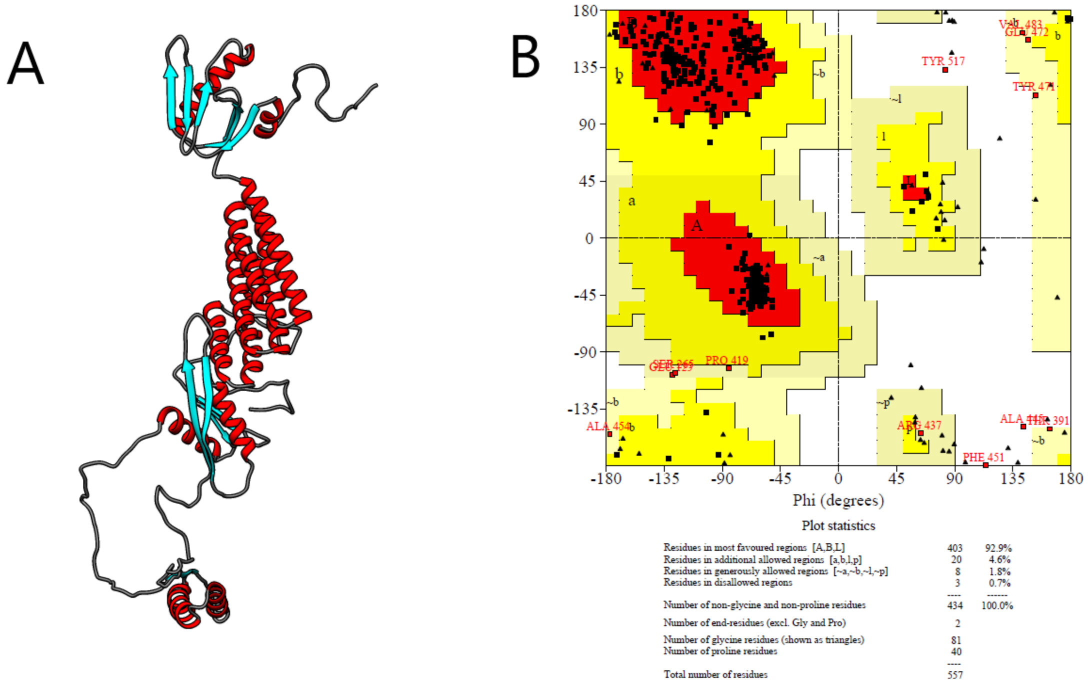

Supplementary Figure S4. Representation of B-lymphocyte conformational epitopes with a score above 0.7 present in the chimeric protein sequence. (Red) 6 residue epitopes, score: 0.969. (Green) 25 residue epitopes, score: 0.898. (Blue) 3 residue epitopes, score: 0.859. (Cyan) 85 residue epitopes, score: 0.745. (Purple) 107 residue epitopes, score: 0.741.

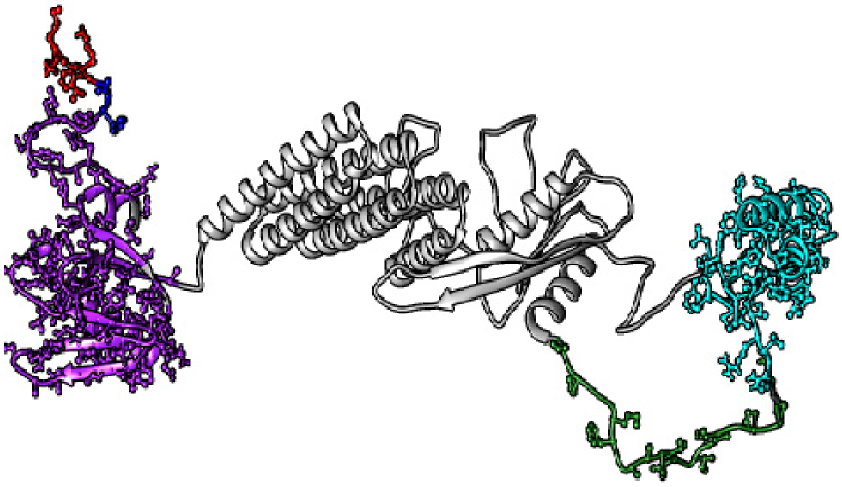

Supplementary Figure S5. Immuno simulation results of only the adjuvant regarding B cell population. (A) B cell population per mm^3 (B) PLB cell population per mm^3.

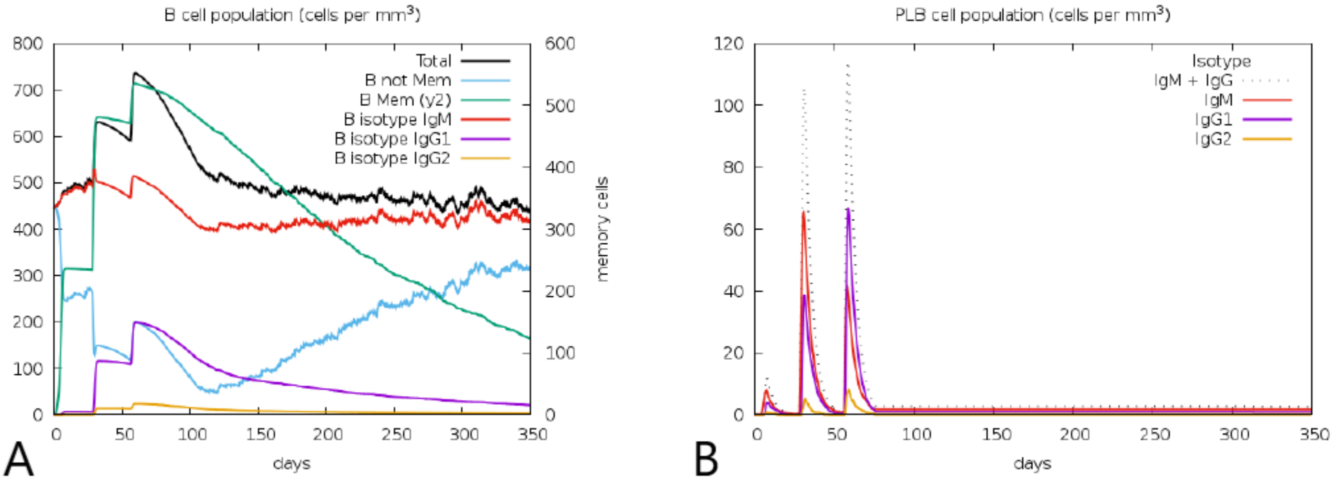

Supplementary Figure S6. Immuno simulation results of only the adjuvant regarding immunoglobulin production.

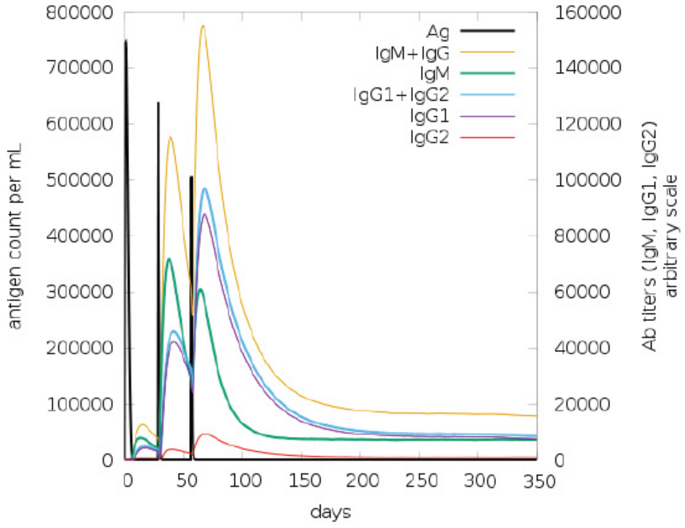

Supplementary Figure S7. Immuno simulation results of only the adjuvant regarding T lymphocyte populations. (A) Helper T-Cell population per state. (B) Helper T-Cell differentiation. (C) Cytotoxic T-Cell population. (D) Cytotoxic T-Cell population per state.

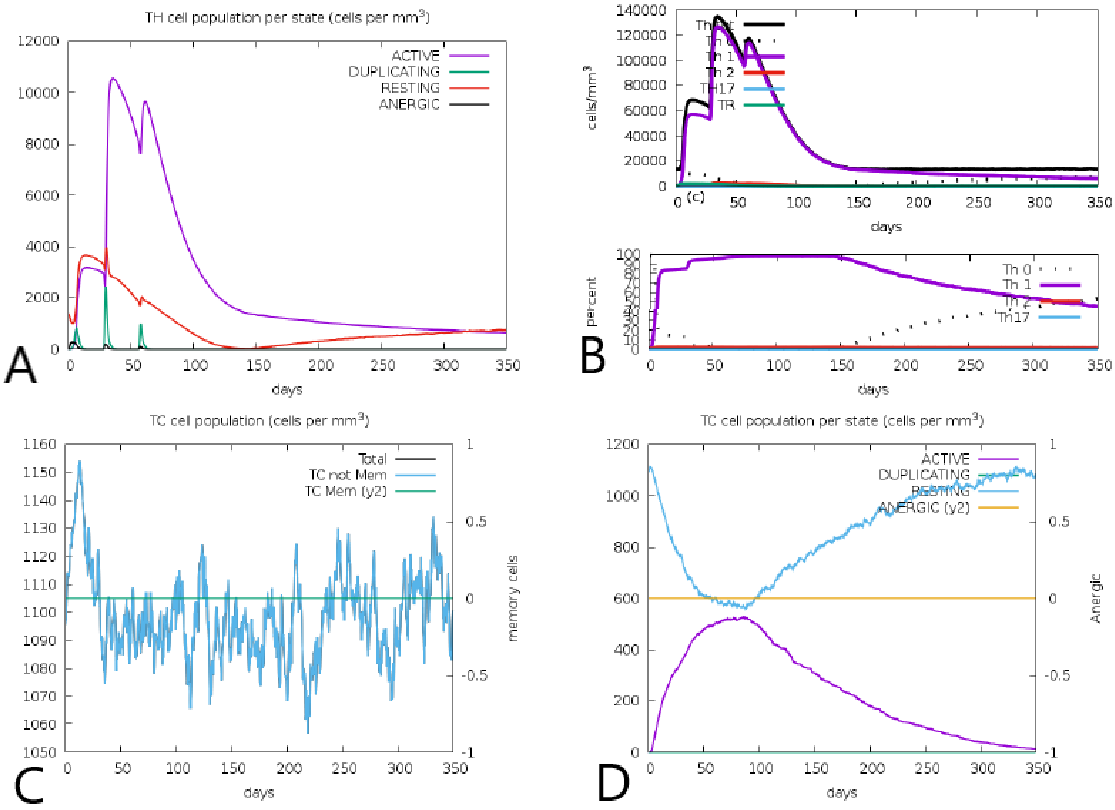

Supplementary Figure S8. Immuno simulation results of the chimeric protein regarding cytokine production.

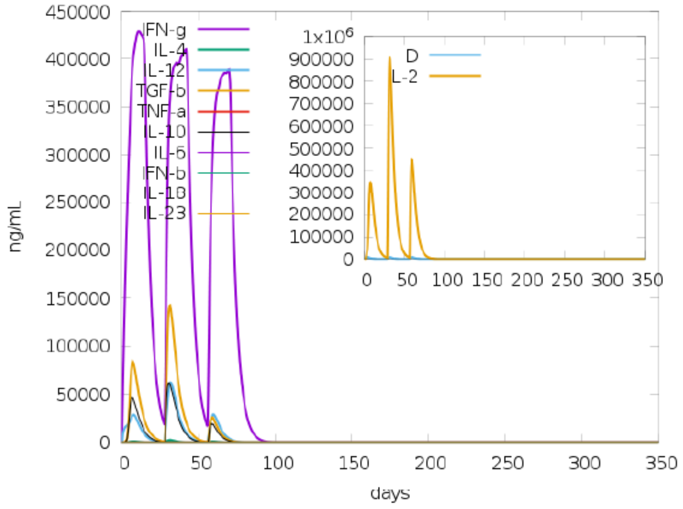

Supplementary Figure S9. Immuno simulation results of only the epitopes regarding B cell population. (A) B cell population per mm^3 (B) PLB cell population per mm^3.

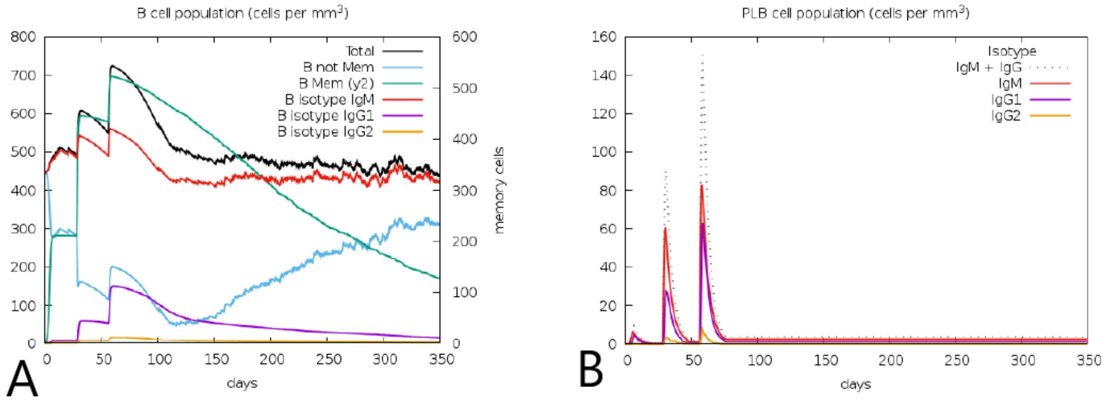

Supplementary Figure S10. Immuno simulation results of only the epitopes regarding immunoglobulin production.

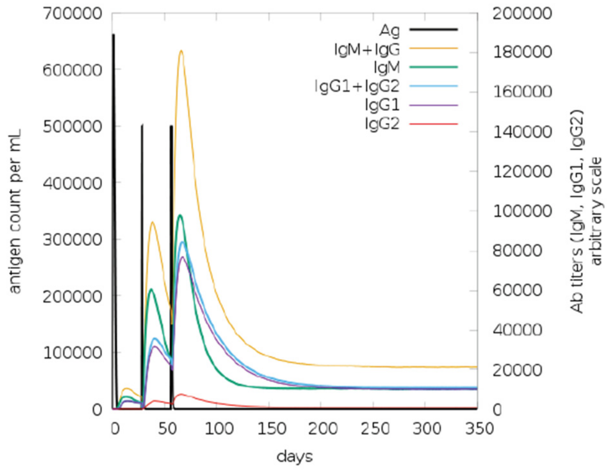

Supplementary Figure S11. Immuno simulation results of only the epitopes regarding T lymphocyte populations. (A) Helper T-Cell population per state. (B) Helper T-Cell differentiation. (C) Cytotoxic T-Cell population. (D) Cytotoxic T-Cell population per state.

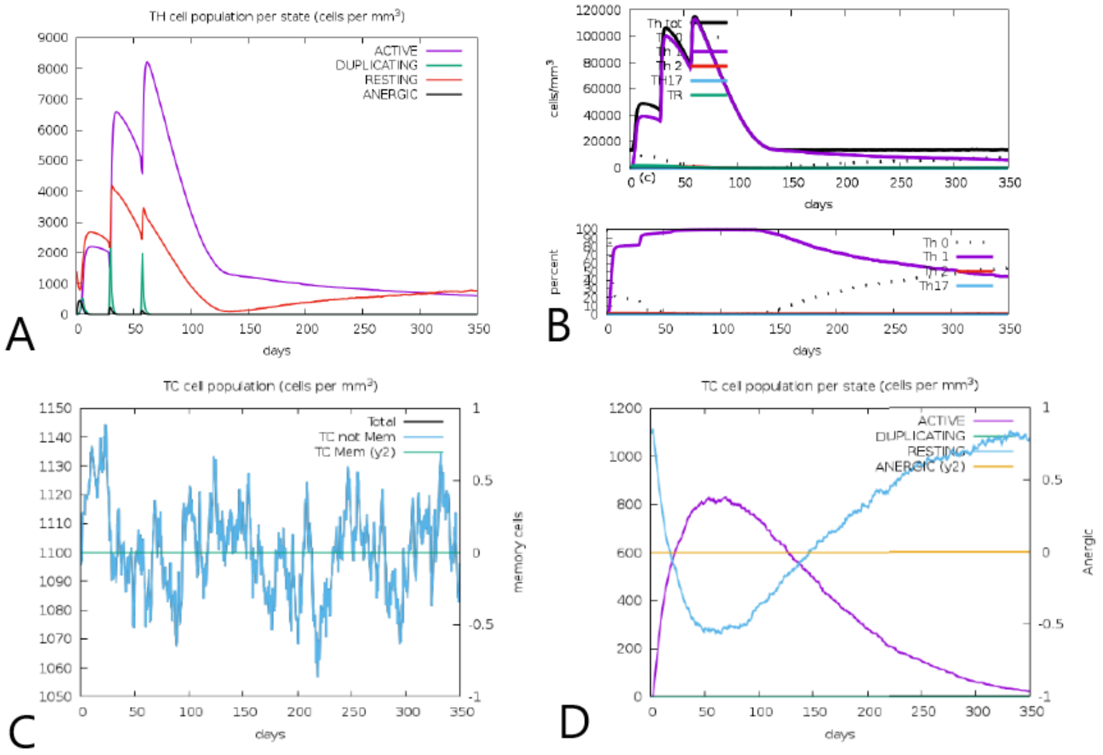

Supplementary Figure S12. Immuno simulation results of the chimeric protein regarding cytokine production.

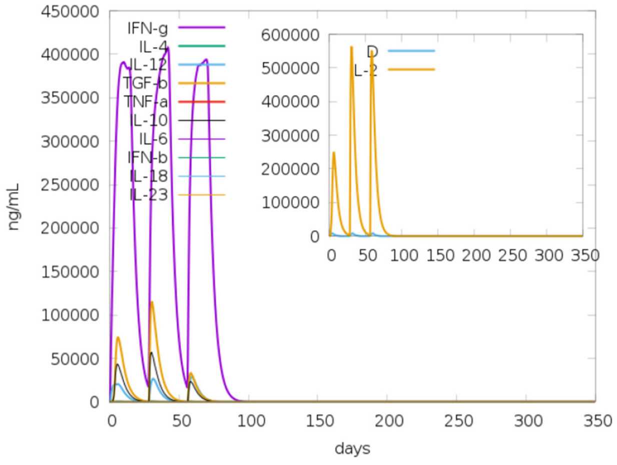

Supplement: Supplementary file 1 [file vaccines-10-01019-s001.zip › vaccines-1763576-supplementary.pdf]
